# Supplementary material for: Watching sports and happiness among older adults in Japan: The JAGES cohort study
Source: PLoS One. 2025 Apr 9;20(4):e0320213. doi: 10.1371/journal.pone.0320213 (PMC11981122; doi:10.1371/journal.pone.0320213)
Supplement: S1 File — S1 Table. Distribution of happiness scores by frequency of watching sports. S2 Table. Comparison of background characteristics between analytic sample and original sample in 2019. S3 Table. Regression coefficients and 95% confidence intervals of linear regression analyses examining the associations between happiness and watching sports in the model, including the two explanatory variables of watching sports on-site and on TV/Internet simultaneously. S4 Table. Regression coefficients and 95% confidence intervals of linear regression analyses examining the associations between happiness and watching sports in the model, including the two explanatory variables of watching professional sports on-site and watching sports on TV/Internet simultaneously. S5 Table. Prevalence ratios and 95% confidence intervals of modified Poisson regression analyses examining the associations between happiness and watching sports in the model, including the two explanatory variables of watching sports on-site and on TV/Internet simultaneously. S6 Table. Prevalence ratios and 95% confidence intervals of modified Poisson regression analyses examining the associations between happiness and watching sports in the model, including the two explanatory variables of watching professional sports on-site and watching sports on TV/Internet simultaneously. S7 Table. Regression coefficients and 95% confidence intervals of linear regression analyses examining the association between happiness and watching sports stratified by sex. S8 Table. Regression coefficients and 95% confidence intervals of linear regression analyses examining the associations between happiness and watching sports stratified by age (< 75 years and ≥ 75 years). (DOCX) [file pone.0320213.s001.docx]

**S1 Table.** **Distribution of happiness scores by frequency of watching sports.**

| **Frequency of watching sports** | **Mean (SD)** |
| --- | --- |
| **Watching sports on-site** |  |
| Never | 7.2 (1.8) |
| A few times a year | 7.6 (1.6) |
| At least once a month | 7.6 (1.8) |
| **Watching professional sports on-site** |  |
| Never | 7.6 (1.7) |
| A few times a year | 7.7 (1.6) |
| At least once a month | 7.3 (1.7) |
| **Watching sports on TV/Internet** |  |
| Never | 7.1 (2.0) |
| A few times a year | 7.2 (1.8) |
| At least once a month | 7.4 (1.7) |

SD: Standard deviation

**S2 Table.** Comparison of background characteristics between analytic sample and original sample in 2019

|  | **Analytic sample** | **Original sample** |
| --- | --- | --- |
| **Characteristic** | **n = 11,265^a^** | **n = 22,528^a^** |
| Sex, female | 5,883 (52.2%) | 11,771 (52.3%) |
| Age (years) | 73.6 (5.6) | 74.5 (6.3) |
| Marital status: married | 8,036 (72.2%) | 16,101 (72.8%) |
| Living arrangements: living alone | 1,455 (13.6%) | 3,350 (15.8%) |
| Occupational status: employed | 3,397 (32.8%) | 6,070 (29.9%) |
| Years of education (years) |  |  |
| < 10 | 2,559 (23.3%) | 5,528 (25.3%) |
| 10–12 | 4,908 (44.7%) | 9,616 (44.0%) |
| ≥ 13 | 3,523 (32.1%) | 6,689 (30.6%) |
| Drinking status: yes | 4,766 (43.8%) | 9,012 (41.6%) |
| Smoking status: yes | 1,059 (9.6%) | 2,250 (10.2%) |
| Equivalent income (yen) |  |  |
| < 2 million | 4,818 (48.7%) | 10,044 (51.4%) |
| 2–4 million | 4,034 (40.8%) | 7,213 (36.9%) |
| > 4 million | 1,041 (10.5%) | 2,300 (11.8%) |
| Body mass index (kg/m^2^) | 23.0 (3.1) | 22.9 (3.2) |
| Instrumental activities of daily living, independent | 10,236 (93.2%) | 19,899 (90.7%) |
| Self-rated health, good | 10,076 (91.5%) | 19,951 (91.5%) |
| Geriatric depression scale (0–15: higher scores indicate a more severe presence of depressive symptoms) | 2.7 (2.8) | 2.9 (3.0) |
| Hypertension, yes | 5,179 (47.3%) | 9,782 (45.3%) |
| Stroke, yes | 245 (2.2%) | 547 (2.5%) |
| Cardiovascular disease, yes | 1,025 (9.4%) | 2,022 (9.4%) |
| Diabetes, yes | 1,645 (15.0%) | 2,977 (13.8%) |
| Dyslipidemia, yes | 1,763 (16.1%) | 3,255 (15.1%) |
| Musculoskeletal disorders, yes | 1,048 (9.6%) | 2,388 (11.0%) |
| Population density (persons/km^2^) |  |  |
| < 1,000 | 4,233 (37.6%) | 7166 (38.7%) |
| 1,000–4,000 | 3,305 (29.3%) | 5168 (27.9%) |
| > 4,000 | 3,727 (33.1%) | 6204 (33.5%) |
| Participation in sports clubs, yes | 3,543 (36.4%) | 6,397 (33.6%) |
| Participation in hobby groups, yes | 4,493 (45.4%) | 8,378 (43.3%) |

^a^n (%); Mean (Standard deviation)

**S3 Table.** **Regression coefficients and 95% confidence intervals of linear regression analyses examining the associations between happiness and watching sports in the model, including the two explanatory variables of watching sports on-site and on TV/Internet simultaneously.**

| **Frequency of watching sports** | **B (95% CI)** | **β** **(95% CI)** | **p-value** |
| --- | --- | --- | --- |
| **Watching sports on-site** |  |  |  |
| Never | Reference | Reference |  |
| A few times a year | 0.11 (0.03, 0.19) | 0.06 (0.02, 0.11) | 0.007 |
| At least once a month | 0.06 (-0.06, 0.19) | 0.04 (-0.03, 0.11) | 0.312 |
| **Watching sports on TV/Internet** |  |  |  |
| Never | Reference | Reference |  |
| A few times a year | -0.04 (-0.12, 0.03) | -0.02 (-0.07, 0.02) | 0.275 |
| At least once a month | 0.02 (-0.05, 0.09) | 0.01 (-0.03, 0.05) | 0.514 |

B: Unstandardized coefficient, β: Standardized coefficient, CI: Confidence interval

Note: All models controlled for sex, age, marital status, living arrangements, occupational status, years of education, drinking status, smoking status, equivalized income, body mass index, instrumental activities of daily living, self-rated health, geriatric depression scale, hypertension, stroke, cardiovascular disease, diabetes, dyslipidemia, musculoskeletal disorders, population density, participation in sports clubs, participation in hobbies, and happiness scores in 2019.

**S4 Table. Regression coefficients and 95% confidence intervals of linear regression analyses examining the associations between happiness and watching sports in the model, including the two explanatory variables of watching professional sports on-site and watching sports on TV/Internet simultaneously.**

| **Frequency of watching sports** | **B (95% CI)** | **β** **(95% CI)** | **p-value** |
| --- | --- | --- | --- |
| **Watching professional sports on-site** |  |  |  |
| Never | Reference | Reference |  |
| A few times a year | 0.12 (0.02, 0.22) | 0.07 (0.01, 0.12) | 0.024 |
| At least once a month | 0.00 (-0.30, 0.30) | 0.00 (-0.17, 0.17) | 0.993 |
| **Watching sports on TV/Internet** |  |  |  |
| Never | Reference | Reference |  |
| A few times a year | -0.04 (-0.12, 0.04) | -0.02 (-0.07, 0.02) | 0.316 |
| At least once a month | 0.03 (-0.04, 0.10) | 0.02 (-0.02, 0.06) | 0.433 |

B: Unstandardized coefficient, β: Standardized coefficient, CI: Confidence interval

Note: All models controlled for sex, age, marital status, living arrangements, occupational status, years of education, drinking status, smoking status, equivalized income, body mass index, instrumental activities of daily living, self-rated health, geriatric depression scale, hypertension, stroke, cardiovascular disease, diabetes, dyslipidemia, musculoskeletal disorders, population density, participation in sports clubs, participation in hobbies, and happiness scores in 2019.

**S5 Table. Prevalence ratios and 95% confidence intervals of modified Poisson regression analyses examining the associations between happiness and watching sports in the model, including the two explanatory variables of watching sports on-site and on TV/Internet simultaneously.**

| **Frequency of watching sports** | **PR** | **95% CI** | **p-value** |
| --- | --- | --- | --- |
| **Watching sports on-site** |  |  |  |
| Never | Reference |  |  |
| A few times a year | 1.07 | 1.03, 1.12 | 0.001 |
| At least once a month | 1.07 | 1.00, 1.14 | 0.042 |
| **Watching sports on TV/Internet** |  |  |  |
| Never | Reference |  |  |
| A few times a year | 0.97 | 0.93, 1.02 | 0.255 |
| At least once a month | 1.00 | 0.96, 1.04 | 0.869 |

CI: Confidence interval, PR: Prevalence ratio

Note: All models controlled for sex, age, marital status, living arrangements, occupational status, years of education, drinking status, smoking status, equivalized income, body mass index, instrumental activities of daily living, self-rated health, geriatric depression scale, hypertension, stroke, cardiovascular disease, diabetes, dyslipidemia, musculoskeletal disorders, population density, participation in sports clubs, participation in hobbies, and happiness scores in 2019.

**S6 Table. Prevalence ratios and 95% confidence intervals of modified Poisson regression analyses examining the associations between happiness and watching sports in the model, including the two explanatory variables of watching professional sports on-site and watching sports on TV/Internet simultaneously.**

| **Frequency of watching sports** | **PR** | **95% CI** | **p-value** |
| --- | --- | --- | --- |
| **Watching professional sports on-site** |  |  |  |
| Never | Reference |  |  |
| A few times a year | 1.06 | 1.01, 1.12 | 0.030 |
| At least once a month | 1.06 | 0.89, 1.26 | 0.526 |
| **Watching sports on TV/Internet** |  |  |  |
| Never | Reference |  |  |
| A few times a year | 0.98 | 0.93, 1.02 | 0.320 |
| At least once a month | 1.00 | 0.96, 1.04 | 0.944 |

CI: Confidence interval, PR: Prevalence ratio

Note: All models controlled for sex, age, marital status, living arrangements, occupational status, years of education, drinking status, smoking status, equivalized income, body mass index, instrumental activities of daily living, self-rated health, geriatric depression scale, hypertension, stroke, cardiovascular disease, diabetes, dyslipidemia, musculoskeletal disorders, population density, participation in sports clubs, participation in hobbies, and happiness scores in 2019.

**S7 Table. Regression coefficients and 95% confidence intervals of linear regression analyses examining the association between happiness and watching sports stratified by sex.**

| **Frequency of watching sports** | **B (95% CI)** | **β** **(95% CI)** | **p-value** |
| --- | --- | --- | --- |
| **Male (n = 5,382)** | | | |
| **Watching sports on-site** |  |  |  |
| Never | Reference | Reference |  |
| A few times a year | 0.16 (0.06, 0.26) | 0.09 (0.04, 0.15) | 0.001 |
| At least once a month | 0.12 (-0.05, 0.28) | 0.07 (-0.03, 0.16) | 0.155 |
| **Watching professional sports on-site** |  |  |  |
| Never | Reference | Reference |  |
| A few times a year | 0.20 (0.08, 0.32) | 0.11 (0.04, 0.18) | 0.002 |
| At least once a month | 0.11 (-0.24, 0.46) | 0.06 (-0.14, 0.26) | 0.527 |
| **Watching sports on TV/Internet** |  |  |  |
| Never | Reference | Reference |  |
| A few times a year | -0.03 (-0.17, 0.10) | -0.17 (-0.09, 0.06) | 0.640 |
| At least once a month | 0.06 (-0.06, 0.17) | 0.03 (-0.03, 0.10) | 0.337 |
| **Female (n = 5,883)** | | | |
| **Watching sports on-site** |  |  |  |
| Never | Reference | Reference |  |
| A few times a year | 0.03 (-0.10, 0.16) | 0.02 (-0.06, 0.09) | 0.655 |
| At least once a month | 0.00 (-0.19, 0.19) | 0.00 (-0.11, 0.11) | 0.976 |
| **Watching professional sports on-site** |  |  |  |
| Never | Reference | Reference |  |
| A few times a year | -0.02 (-0.20, 0.16) | -0.01 (-0.11, 0.09) | 0.846 |
| At least once a month | -0.19 (-0.74, 0.35) | -0.10 (-0.41, 0.21) | 0.492 |
| **Watching sports on TV/Internet** |  |  |  |
| Never | Reference | Reference |  |
| A few times a year | -0.04 (-0.13, 0.06) | -0.02 (-0.07, 0.03) | 0.447 |
| At least once a month | 0.02 (-0.07, 0.11) | 0.01 (-0.04, 0.06) | 0.633 |

B: Unstandardized coefficient, β: Standardized coefficient, CI: Confidence interval

Note: All models controlled for sex, age, marital status, living arrangements, occupational status, years of education, drinking status, smoking status, equivalized income, body mass index, instrumental activities of daily living, self-rated health, geriatric depression scale, hypertension, stroke, cardiovascular disease, diabetes, dyslipidemia, musculoskeletal disorders, population density, participation in sports clubs, participation in hobbies, and happiness scores in 2019.

**S8 Table. Regression coefficients and 95% confidence intervals of linear regression analyses examining the associations between happiness and watching sports stratified by age (< 75 years and ≥ 75 years).**

| **Frequency of watching sports** | **B (95% CI)** | **β** **(95% CI)** | **p-value** |
| --- | --- | --- | --- |
| **Age < 75 (n = 6,653)** | | | |
| **Watching sports on-site** |  |  |  |
| Never | Reference | Reference |  |
| A few times a year | 0.10 (0.00, 0.20) | 0.06 (0.00, 0.11) | 0.046 |
| At least once a month | 0.14 (-0.03, 0.30) | 0.08 (-0.01, 0.17) | 0.107 |
| **Watching professional sports on-site** |  |  |  |
| Never | Reference | Reference |  |
| A few times a year | 0.14 (0.01, 0.27) | 0.08 (0.01, 0.15) | 0.030 |
| At least once a month | -0.15 (-0.54, 0.24) | -0.09 (-0.31, 0.14) | 0.445 |
| **Watching sports on TV/Internet** |  |  |  |
| Never | Reference | Reference |  |
| A few times a year | 0.04 (-0.06, 0.13) | 0.02 (-0.03, 0.08) | 0.428 |
| At least once a month | 0.06 (-0.03, 0.14) | 0.03 (-0.02, 0.08) | 0.202 |
| **Age ≥ 75 (n = 4,612)** | | | |
| **Watching sports on-site** |  |  |  |
| Never | Reference | Reference |  |
| A few times a year | 0.13 (-0.00, 0.26) | 0.07 (0.00, 0.14) | 0.050 |
| At least once a month | -0.01 (-0.20, 0.18) | 0.00 (-0.11, 0.10) | 0.920 |
| **Watching professional sports on-site** |  |  |  |
| Never | Reference | Reference |  |
| A few times a year | 0.09 (-0.08, 0.26) | 0.05 (-0.04, 0.14) | 0.278 |
| At least once a month | 0.14 (-0.32, 0.60) | 0.08 (-0.17, 0.33) | 0.543 |
| **Watching sports on TV/Internet** |  |  |  |
| Never | Reference | Reference |  |
| A few times a year | -0.15 (-0.28, -0.02) | -0.08 (-0.15, -0.01) | 0.028 |
| At least once a month | 0.02 (-0.10, 0.13) | 0.01 (-0.05, 0.07) | 0.750 |

B: Unstandardized coefficient, β: Standardized coefficient, CI: Confidence interval

Note: All models controlled for sex, age, marital status, living arrangements, occupational status, years of education, drinking status, smoking status, equivalized income, body mass index, instrumental activities of daily living, self-rated health, geriatric depression scale, hypertension, stroke, cardiovascular disease, diabetes, dyslipidemia, musculoskeletal disorders, population density, participation in sports clubs, participation in hobbies, and happiness scores in 2019.
