# Supplementary material for: Watching sports and happiness among older adults in Japan: The JAGES cohort study
Source: PLoS One. 2025 Apr 9;20(4):e0320213. doi: 10.1371/journal.pone.0320213 (PMC11981122; doi:10.1371/journal.pone.0320213)
Supplement: Attachment — Submitted filename: response_letter.docx [file pone.0320213.s003.docx]

Dear Editor,

We sincerely thank the reviewers for their careful evaluation of our manuscript and their constructive comments. We have addressed all comments and revised our manuscript accordingly. Below are our point-by-point responses.

**Response to Reviewer #1:**

**Comment 1: The references cited in the introduction and discussion sections only go up to the year 2022. This could lead professional peers to believe that there is a lack of literature on the topic in the most recent years, 2023-2024.**

**Comment 2: Has there been any recent attention from peers in the past two years regarding the relationship between passive sports participation and the happiness of older adults?**

Response: We appreciate this valuable suggestion. We have conducted an additional literature review to include the most recent studies from 2023-2024 and added some new insights into online sports watching and biological mechanism.

Changes: We have updated references in our introduction. The list of new references is provided as follows:

• Yoshida M, Sato M, Doyle J. Feeling vital by watching sport: The roles of team identification and stadium attendance in enhancing subjective vitality. J Sport Manag. 2023;37: 229-242. doi: 10.1123/jsm.2021-0174.

• Kinoshita K, Matsuoka H. Risk and psychological return: a moderating role of COVID-19 risk perception in the impact of team identification on vitality after sport spectatorship in Tokyo. Int J Sports Mark Spons. 2023;24: 20-37. doi: 10.1108/IJSMS-01-2022-0007.

• Lin Y-H, Chen C-Y, Lin Y-K, Lee C-Y, Cheng C-Y. Effects of Online Video Sport Spectatorship on the Subjective Well-Being of College Students: The Moderating Effect of Sport Involvement. Int J Environ Res Public Health. 2023; 20:4381. doi: 10.3390/ijerph20054381.

• Kinoshita K, Nakagawa K, Sato S. (2024). Watching sport enhances well-being: evidence from a multi-method approach. Sport Manage Rev. 2024;27: 595-619. doi: 10.1080/14413523.2024.2329831

We have modified the introduction section with recent references:

“Previous studies have reported that passive sports participation is associated with greater subjective well-being [10-12]” (p.4, line 65).

“According to a study focusing on college students [19], online video sport spectatorship may improve subjective well-being, with sport participation moderating the relationship between online video sport spectatorship and well-being.” (p. 4, lines 78–81).

We have updated the discussion section with recent references:

“In terms of biological mechanisms, watching popular sports increases brain activity in regions associated with positive emotions, leading to enhanced self-reported well-being [34].” (p. 24, lines 300-302).

**Comment 3: The discussion of two counterintuitive findings (frequency of watching and non-participation in sports clubs) could be more exhaustive.**

**Response:** Thank you for this valuable suggestion. We appreciate the reviewer's insight that these counterintuitive findings require more detailed explanation. We have expanded our discussion of these findings with additional theoretical frameworks and supporting evidence.

**Changes:** We have modified the text in the discussion (p. 24–25, lines 308–322): “While frequent sports watching might intuitively seem more beneficial, moderate frequency (a few times per year) appears optimal for happiness. The complex relationship between watching frequency and happiness can be explained by three mechanisms. First, regular spectators often experience stronger negative emotions from losses, which can offset positive effects [35]. This emotional variability may explain why moderate frequency (a few times per year) shows optimal effects on happiness. Second, a study on the connection between watching sports and well-being [36] indicated that an individual’s psychological connection with a sports team may be more important than watching frequency itself for greater well-being. The relationship between watching sports and happiness may depend more on how much attachment individuals have to their favorite teams rather than how often they watch sports. Third, the hedonic adaptation theory suggests that people tend to return to a baseline level of happiness despite repeated positive experiences [37]. Frequent sports watching may lead to habituation, reducing its emotional effect over time. Thus, the relationship between sports watching and happiness appears to be influenced by both quantitative and qualitative aspects of sports engagement.”

We have updated the following text in the discussion (p. 26, lines 337–351): “The effect of watching sports on happiness may vary depending on older adults' active participation in sports. Many studies have consistently demonstrated that active participation in sports can lead to higher happiness levels [9]. However, it is generally not easy to encourage older adults, especially those with functional decline or limited mobility, to engage in regular sports or exercise. Our findings suggest that watching sports may have different implications for participants versus non-participants in sports clubs. Sports club participants may already receive social and emotional benefits through their active participation, creating a ceiling effect for additional benefits from watching sports. In contrast, non-participants may experience unique social connection and sports-related enjoyment primarily through watching sports. Additionally, the novelty effect may be stronger for non-participants. While sports club participants are regularly exposed to sports environments, individuals who do not participate in sports may find the atmosphere and excitement of live sports events more stimulating and beneficial on their emotional state. However, these potential mechanisms remain hypothetical, and future research is needed to examine the psychological processes underlying the differential effects of sports watching between sports participants and non-participants.”

**Response to Reviewer #2:**

**Comment 1: Abstract must be clearly about the aim of the study; it seems to be different in the text.**

**Response:** Thank you for this important observation regarding the consistency of our study aims. We have revised the abstract to ensure consistency between the stated aims in the abstract and main text.

**Changes:** The objective statement in the abstract (p. 2, lines 22–24) was modified to read: “This study aimed to investigate the association between different forms of sports spectatorship (on-site and on TV/Internet) and happiness among older adults in Japan.”

**Comment 2: In the method, were possible to have an instrumentation item and another one to describe the population.**

**Response:** Thank you for this constructive suggestion to improve the organization of our methods section. We have restructured the methods section to include separate subsections for instrumentation and population description.

**Changes:** Added two new subsections in the methods:

1. “Study population” (p. 6, lines 105-112): “Between November and December 2022,...”

2. “Measures” (p. 6–7, lines 113-160): “In our research,...”

**Comment 3: In the results, there will be possible to highlight the results of the Happiness score (maybe in a separate table).**

**Response:** Based on your suggestion, we have created a new table specifically focusing on happiness scores and their distribution across different viewing patterns.

**Changes:** Added S1 Table: “Distribution of happiness scores by frequency of watching sports.” (Supporting information) and the following text in the results (p.10, lines 201–208):

“S1 Table shows the distribution of happiness scores by frequency of watching sports. Participants who watched sports on-site over a few times a year reported higher happiness scores (mean = 7.6) than those who never watched sports (mean = 7.2). Regarding professional sports on-site, happiness scores were similar between those who never watched (mean = 7.6) and those who watched a few times a year (mean = 7.7). Participants who watched sports on TV/Internet at least once a month showed higher happiness scores (mean = 7.4) than those who never watched (mean = 7.1).”

**S1 Table.** **Distribution of happiness scores by frequency of watching sports.**

| **Frequency of watching sports** | **Mean (SD)** |
| --- | --- |
| **Watching sports on-site** |  |
| Never | 7.2 (1.8) |
| A few times a year | 7.6 (1.6) |
| At least once a month | 7.6 (1.8) |
| **Watching professional sports on-site** |  |
| Never | 7.6 (1.7) |
| A few times a year | 7.7 (1.6) |
| At least once a month | 7.3 (1.7) |
| **Watching sports on TV/Internet** |  |
| Never | 7.1 (2.0) |
| A few times a year | 7.2 (1.8) |
| At least once a month | 7.4 (1.7) |

SD: Standard deviation

**Comment 4: Some suggestions must be done with the specific purpose, with the findings, suggestions to increase happiness of these specific population.**

**Response:** We appreciate your suggestion regarding the need for practical implications derived from our findings. We have added specific recommendations based on our findings.

**Changes:** We have modified the text in the discussion (p. 27, lines 370–381): “Our research reveals a positive association between watching sports on-site and happiness, but not with TV or Internet watching. Our findings have several implications. First, it suggests the importance of creating environments that promote watching sports on-site rather than on TV or Internet. While watching sports on TV or the Internet is accessible, visiting stadiums or arenas for spectatorship may be more effective in enhancing happiness [40]. Second, optimizing the use of existing facilities, such as public parks and stadiums, could help promote happiness among older adults [46]. These venues could be designed to facilitate the social aspects of live sports events that may not be obtained through TV or Internet watching. Third, providing older adults with discounted or free tickets to sports events could serve as an effective population-based strategy for promoting happiness [18]. Overall, initiatives that encourage on-site sports spectatorship could play a key role in improving happiness among older adults.”
