# Supplementary material for: Watching sports and happiness among older adults in Japan: The JAGES cohort study
Source: PLoS One. 2025 Apr 9;20(4):e0320213. doi: 10.1371/journal.pone.0320213 (PMC11981122; doi:10.1371/journal.pone.0320213)
Supplement: Attachment — Submitted filename: response_letter_auresp_3.docx [file pone.0320213.s005.docx]

Dear Reviewers,

Thank you very much for your insightful and constructive feedback on our manuscript. We appreciate the time and effort you have dedicated to helping us improve our work. We have carefully considered each of your comments and have revised the manuscript accordingly. Below, we provide a detailed response to each point raised.

**Methodology**

#1. The sampling methodology and the criteria for inclusion and exclusion require more comprehensive elucidation.

#2. Elucidate the methods employed for participant recruitment and the justification for the inclusion and exclusion criteria established. This is especially significant considering that approximately fifty percent of the original participants were eliminated, which raises issues regarding attrition bias and the generalizability of the findings.

#3. Specify the methods employed to contact participants (e.g., via community centers, health clinics, or online platforms).

#4. Elucidate the techniques for distributing questionnaires (e.g., postal surveys, face-to-face interviews, or electronic forms) to offer insights into response rates and any biases.

**Response:** We sincerely thank the reviewers for their valuable feedback regarding the methodology and the potential implications of sample attrition. We have addressed these points comprehensively in the revised manuscript and provide a detailed response below. We have expanded the Methods section to provide a more thorough description of the JAGES study design. To address the potential for attrition bias, we conducted a detailed comparison of the background characteristics between our analytic sample and the original 2019 sample.

Lines 89–102:
“Data were obtained from the Japan Gerontological Evaluation Study (JAGES) [21], an ongoing cohort study examining social factors influencing the health of community-dwelling individuals aged 65 years and older. Since 2003, JAGES surveys have been conducted approximately every three years in collaboration with municipal governments across Japan [21]. These governments develop and implement long-term care insurance plans, including preventive care services. JAGES surveys provide critical data for assessing policy effectiveness and guiding future planning [21]. Within each municipality, eligible individuals aged 65 years and older without long-term care certification are selected from the resident registry. In small municipalities, all eligible older adults are included, whereas in large municipalities, 30% of the target sample is randomly selected. Among the remaining 70%, individuals aged 65 to 67 years are chosen proportionally based on population distribution. Subsequently, individuals aged 68 years and older who participated in the most recent survey are prioritized for recruitment. Additional participants aged 68 years and older are enrolled until the target sample size is achieved.”

Lines 123–126:

“The JAGES 2022 survey, conducted between November and December 2022, mailed questionnaires containing items related to sports spectatorship to 42,096 community-dwelling individuals aged ≥ 65 years, identified through official residential registers provided by municipal governments.”

#5. Elucidate the methodology employed to assess ADL independence. Indicate if validated instruments or standardized scales were employed and their significance to the study context.

**Response:** Thank you for raising this point. We have expanded the Methods section to further clarify that ADL independence was assessed through a self-reported question: "Do you require nursing care or assistance in daily life from anyone?"

Lines 129–130:

“Lack of independence in ADLs was defined based on self-reported responses to the question, ‘Do you require nursing care or assistance in daily life from anyone?’”

#6. Summarize the Japanese short version of the Geriatric Depression Scale (GDS), detailing its objective, framework, and methodology for assessing depression symptoms. Emphasize the scale's validity and reliability to substantiate its application in the research.

**Response:** Thank you for the suggestion. We have provided a more detailed description of the Japanese short version of the GDS in the Methods section. The revised text now includes evidence of its validity and reliability in assessing depressive symptoms among older adults in Japan.

Lines 172–176:

“The Japanese short version of the Geriatric Depression Scale (GDS) was used to assess depressive symptoms. This 15-item scale, validated for older adults in Japan, has demonstrated good reliability (Cronbach’s α = 0.80) and sensitivity (96%) and specificity (95%) for detecting major depressive disorder [26]. Scores range from 0 to 15, with higher scores indicating more severe depressive symptoms.”

**Results and Statistical Analysis**

#7. The elimination of about fifty percent of the subjects substantially affects the study's generalizability. Identify potential obstacles associated with this issue and offer arguments or techniques for alleviating attrition bias.

**Response:** We acknowledge this limitation. We have performed a comparison between our analytic sample and the original sample to assess potential selection bias (reported in S2 Table and discussed in the Results section).

Lines 240–244:
“To assess potential selection bias from sample attrition, we compared our analytic sample with the original sample in 2019 (n=22,528; S2 Table). The background characteristics of our analytic sample were largely comparable to those of the original sample, although our analytic samples included slightly younger participants (mean age: 73.6 vs 74.5 years).”

#8. Explore alternate metrics for happiness, such ordinal regression or continuous happiness scores, in additional analysis to maintain information integrity and evaluate the robustness of dichotomized outcomes.

**Response:** We appreciate this suggestion and have already performed linear regression using happiness scores as a continuous variable. The results were consistent with the dichotomized outcome analysis, ensuring the robustness of our findings. We have added this detail to the manuscript, along with a discussion of its implications. By using both continuous and dichotomized outcomes, we provide a comprehensive evaluation of the relationship between sports spectatorship and happiness.

#9. Directly tackle multicollinearity concerns, especially within multivariable models.

**Response:** Thank you for raising this concern. We assessed multicollinearity by calculating the Variance Inflation Factor (VIF) for each model. All VIF values were less than 10, indicating no significant multicollinearity. We have explicitly stated this in the Methods section of the revised manuscript.

Lines 212–214:

“To assess multicollinearity, we calculated the Variance Inflation Factor (VIF) for each model. All VIFs values were less than 10, indicating no significant multicollinearity in the models.”

#10. Enhance transparency by disclosing subgroup sizes and addressing restrictions arising from small samples or diminished statistical power.

**Response:** We have ensured that the sizes of all subgroups are clearly reported in the Results section and relevant tables.

Lines 314–317, 322, 327

“In the subgroup analysis of participation in sports clubs at least a few times a year (n = 4,091), none of the three forms of watching sports was associated with a higher happiness score (as presented in Table 5). However, the subgroup analysis of non-participation in sports clubs (n = 7,174)”

“In the sex-stratified analysis (male: n = 5,382; female: n = 5,883),”

“In the age-stratified analysis (< 75 years: n = 6,653; ≥ 75 years: n = 4,612),”

#11. Incorporate standardized coefficients to offer detailed insights into the comparative influence of various forms of sports viewing on happiness.

**Response:** We appreciate your suggestion. We have reported both unstandardized and standardized coefficients.

**Discussion**

#12. Elucidate the impact of various forms of sports viewership on happiness and assess how these results correspond with or contrast to the current body of knowledge.

**Response:** We appreciate the reviewer's insightful suggestion to further elucidate the differential impact of various forms of sports viewership on happiness. We have revised the Discussion section to explicitly highlight the contrasting of on-site versus TV/Internet spectatorship. Specifically, we have modified the text to emphasize that the observed positive association between watching sports and happiness was unique to on-site spectatorship and was not found for TV/Internet viewing.

Lines 406–407

“The positive association between watching sports and happiness was specific to on-site spectatorship and not observed with TV/Internet watching.”

#13. Recognize the constraints on the generalizability of findings stemming from the elevated exclusion rate and the particular demographic concentration on Japanese older persons.

#14. Elaborate on potential biases and constraints, and provide avenues for future research, such the replication of the study in other populations or the utilization of longitudinal designs.

**Response:** Thank you for your suggestion. We have explicitly acknowledged these limitations in the revised manuscript and expanded the Limitations section.

Lines 474–491

“This study has several limitations. First, generalizability is limited by our focus on community-dwelling older adults in Japan. Cultural factors may influence the relationship between sports spectatorship and happiness, warranting caution when extending these findings to other populations. Second, while our analytic sample was largely comparable to the original sample, the sample attrition could have introduced selection bias, potentially skewing the sample towards more active individuals. Additionally, although we adjusted for several confounders, unmeasured variables such as sporting event type, motivation for attendance, and team preferences were not considered due to questionnaire constraints [47]. The COVID-19 pandemic may also have affected our data, as concerns about infection could have diminished enjoyment and attenuated feelings of happiness among spectators [48]. Furthermore, the primarily cross-sectional design restricts our ability to infer causality. Although we attempted to reduce reverse causation by using covariates measured prior to exposure, we cannot eliminate the possibility that happier individuals are simply more inclined to watch sports. Future research should address these limitations by replicating this study in diverse populations across different cultures. Longitudinal designs are needed to establish temporal relationships and elucidate the dynamic interplay between sports spectatorship and happiness. Further investigation into underlying mechanisms (e.g., social interaction, emotional responses, team identification) and the moderating effects of sport type and viewing context is also warranted.”
